# Stigma and Barriers to Mental Health and Substance Use Care for Hispanic/Latine Individuals Living with HIV or at Risk for HIV in Texas, United States

**DOI:** 10.3390/ijerph23060721

**Published:** 2026-05-28

**Authors:** Roxana Guzman, Daniel Castellanos, Evelio Salinas Escamilla, Sana Malik, Melissa Bessaha, Miguel Muñoz-Laboy

**Affiliations:** 1 Latino Commission on AIDS, Houston, TX 77036, USA; rguzman@latinoaids.org (R.G.); esescamilla@latinoaids.org (E.S.E.); 2 Latino Commission on AIDS, New York, NY 10010, USA; dcastellanos@latinoaids.org; 3 School of Social Welfare, Stony Brook University, Stony Brook, NY 11794, USA; sana.k.malik@stonybrook.edu (S.M.); melissa.bessaha@stonybrook.edu (M.B.)

**Keywords:** Latines, Hispanics, barriers to care, HIV care, mental health stigma, substance use related stigma, community health centers, cross-sectional survey, Texas

## Abstract

**Highlights:**

**Public health relevance—How does this work relate to a public health issue?**

- Hispanic/Latine individuals with HIV or at risk for HIV experience profound disparities in access to mental health and substance use treatment services, resulting in persistent inequities in mental health and substance use outcomes.
- Hispanic/Latine populations are the largest and fastest-growing immigrant group in the United States. Their overall health and mental health are adversely affected by systemic inequities, often driven by stigma and discrimination.

**Public health significance—Why is this work of significance to public health?**

- Stigma plays a major role in creating barriers to mental health and substance use services; however, limited data exist on how these factors are connected within Hispanic/Latine communities.
- Identifying the demographic profiles of Hispanic/Latine individuals with HIV or at high-risk for HIV who experience high levels of barriers to substance use and mental health services should be a public health priority.

**Public health implications—What are the key implications or messages for practitioners, policy makers, and/or researchers in public health?**

- Barriers to substance use and mental health services were associated with specific demographic and health-related factors, suggesting areas for programmatic prioritization in community health centers. In particular, younger individuals and women faced more barriers than other subgroups.
- Levels of substance use and mental health stigma were moderate to high in the sample, indicating that continued efforts to address multiple forms of stigma should remain a priority for health centers.

**Abstract:**

Objectives: To describe levels of barriers to mental health and substance use services and to examine the associations, if any, between these barriers and substance use and mental health stigma among Hispanic/Latine adults receiving services for HIV prevention or HIV treatment. Methods: We conducted a cross-sectional survey as part of a mixed-method, community-based statewide study among Hispanic/Latine individuals served by HIV service delivery organizations in Texas, United States, between September 2022 and May 2023 (n = 300). Participants completed a computer-based survey at collaborating organizations. Analyses included descriptive statistics and linear regression modeling. Results: Demographic and background characteristics were significantly associated with differences in reported barriers to mental health and substance use services. Mental health-related stigma was significantly associated with barriers to mental health services. Conclusions: Strategies to reduce barriers, tailored to specific demographic subgroups, and interventions to address mental health-related stigma should be prioritized by HIV service delivery organizations serving Hispanic/Latine communities.

## 1. Introduction

Hispanic/Latine populations in the United States comprise recent migrants, transnational migrants, long-term migrants, and multigenerational communities established prior to the formation of modern U.S. borders, including those predating the Treaty of Guadalupe Hidalgo. Despite this heterogeneity, Hispanic/Latine populations consistently report higher levels of internalized and enacted stigma related to mental illness and substance use, as well as lower utilization and retention in behavioral health services compared to their White, non-Hispanic counterparts [1,2]. These disparities are likely more pronounced in states such as Texas, which have large populations of both recent migrants and long-standing multigenerational Hispanic/Latine communities [3,4]. Furthermore, Texas ranks 47th in state-level expenditures on health and mental health services [3].

In 2021, the Hispanic Health Network (HHN), an alliance of health organizations in Texas, commissioned the identification of multilevel barriers to mental health and substance use treatment among Hispanic/Latine individuals served by HIV prevention and treatment organizations. Mental health- and substance use-related stigma have been associated with negative perceptions of care, which in turn influence individual physical and mental health outcomes among those experiencing mental health and substance use disorders [5]. However, there is limited empirical evidence examining whether—and to what extent—negative perceptions of treatment increase the likelihood of encountering barriers when attempting to access care [5]. Thus, the objectives of this assessment were to: (1) identify multilevel barriers to care across demographic profiles of Hispanic/Latine clients/patients; (2) examine associations between barriers and internalized negative perceptions related to seeking mental health and substance use services.

### Conceptual Framework

This assessment was guided by Williams’ (2013) Racism and Health framework, which conceptualizes barriers to care as products of systemic racism [6,7]. Within this framework, structural inequities contribute to experienced and internalized stigma, which in turn reinforce perceived barriers to accessing mental health and substance use services [6,7]. Under this framework, the weathering of structural racism for minoritized groups, such as recent and long-term migrant Latine/Hispanic communities in the United States, results in the social production of perceived barriers to care based on individual and community-level experiences of discrimination [6,7]. Moreover, experiencing external barriers in access to care, such as insurance, cultural beliefs contradictory to mental health or substance use services, and institutional issues in accessing services, by the individuals or others in their social networks, reinforced individuals’ perceptions and likelihood of accessing services [6,7]. Understanding perceived and external barriers is critical for health services organizations to improve their efforts in increasing access and retention in services, such as substance use and mental health care.

## 2. Materials and Methods

### 2.1. Study Design

A cross-sectional survey was developed using a community-based collaborative approach. Survey items were generated through seven focus groups with representatives from HHN organizations. The instrument was refined by the study authors and further reviewed during two additional consortium meetings. Prior to implementation, each organization assessed the survey for cultural and contextual appropriateness. Data were collected between January and May 2023. Participants were recruited from six HHN-affiliated, multi-service HIV/STI, mental health, and social service organizations located across the Texas State Department of Health Services’ public health regions (Houston, Fort Worth, San Antonio, Lubbock, El Paso, and Harlingen/McAllen, TX, USA).

### 2.2. Measures

The survey consisted of three parts: systemic barriers to mental health and substance use care; negative perceptions of mental health and substance use services; and patient demographic characteristics.

Multilevel barriers to mental health and substance use care: Multilevel barriers were conceptualized by the HHN community consensus. Respondents were asked the following: “In your opinion, how significant are the following factors in preventing Hispanics from utilizing [alcohol/substance services] or [mental health services]?. In this survey “Hispanics” refer to Hispanics, Latines or people from Latin American ancestry.” The list of answer items was generated from providers and clients’ perceived and experienced barriers in four areas: personal, insurance, institutional, and cultural barriers to seek care in two areas: mental health and substance use treatment and care. Perceived personal barriers to seeking services were assessed through 8 items [e.g., “Hispanics lack overall knowledge of alcohol or substance abuse; Hispanics lack the skills to recognize the signs and symptoms of depression, anxiety or post-traumatic stress disorder; Hispanics lack the knowledge to manage their own mental health concerns”] under each domain (substance use and mental health). Insurance barriers were assessed through 5 items [“Hispanics don’t have insurance coverage for substance use services; Hispanics had negative experiences accessing these services; Hispanics do not have the time because of work or family responsibilities”] under each domain (substance use and mental health). Institutional barriers to seeking services were assessed through 11 items [“Services are not available at all in the area where Hispanics live; There is a long wait to access these services; Services are not available in the preferred language”] under each domain (substance use and mental health). Cultural barriers to seeking services were assessed through 4 items [“Hispanics feel that their alcohol or substance use is not an issue or severe enough; Hispanics feel that they can manage their alcohol or substance use on their own; Hispanics prefer to talk with their friends or religious or spiritual advisors”] under each domain (substance use and mental health). For each item, respondents ranked their answers in a 4-point Likert significance scale (1 = not significant, 2 = somewhat significant, 3 = significant, and 4 = very significant).

Each of the above domains was combined to create composite measures of barriers, with the higher the value indicating a higher level of significance to the respondent. The Cronbach’s Alpha coefficients for each of the above composite scales ranged from 0.70 to 0.85.

Internalized negative attitudes towards mental health and substance use services: The HHN group decided to use standardized measures to measure internalized negative attitudes towards mental health and substance use services. These served as the outcome variables for the data analysis. Two scales were used: (1) Perceived Stigma of Substance Abuse Scale, PSAS, consisting of composite scale of 8 items, where higher values signify higher agreement (Mean for this sample: 20.09, SD: 3.38, Cronbach’s Alpha coefficient, CA: 0.73); (2) Perceived Devaluation and Discrimination Scale, PDD, consisting of composite scale of 8 items, where higher values signify higher agreement (Mean: 22.58, SD: 3.71, Cronbach’s Alpha coefficient, CA: 0.81 [8,9,10].

Demographic and background characteristics: The third component of the survey focused on the following characteristics: (1) birth place, (2) gender identity, (3) sexual orientation, (4) highest level of education completed, (5) age, (6) race (based on US census categories), (7) Hispanic/Latine identity, and, (8) current mental or physical health diagnoses [presence/absence of any anxiety disorder, post-traumatic stress disorder (PTSD), any depressive disorder, alcohol use disorder, any substance use disorders (SUD), Hepatitis C, and/or HIV].

### 2.3. Procedures

Surveys were distributed in controlled settings within the organization using Chromebooks to take the survey online or through links sent to the organization’s corresponding list of clients. The survey, available in English and Spanish for linguistic appropriateness, took, on average, between 18 and 20 min to complete, and participants received a $25 electronic gift card incentive to a major full-line supermarket with a discount department store, offering a broad selection of groceries, electronics, apparel, household goods, and automotive products.

### 2.4. Participants and Sample Size

A total of 375 Hispanic/Latine patients/clients were approached (n~65 × 6 organizations). The intended sample size (n = 300) was achieved with a rejection rate close to 20% across all organizations, primarily due to not having time to complete the survey.

### 2.5. Data Analysis

We conducted descriptive statistics on all study variables. Our analysis was divided into two parts. We first used a series of independent *t*-tests to determine if there were statistically significant differences in barriers’ average scores by subgroups based on demographic and health characteristics (e.g., institutional barriers score differences between those with a depression diagnosis compared to those without a diagnosis). Given that this was a community-based, cross-sectional study, we conducted exploratory analyses to examine the associations, if any, between stigma scores and the composite score of barriers. We decided to test eight equations, with Y’ as stigma scores (PSAS or PDD), X_1_ as the composite score for each type of barrier, and X_2_ as age. We decided on age and birthplace as the primary controlling-adjusting variables. Age and X_3_, as place of birth (foreign vs. US-born), were selected because both are highly relevant for HIV programming. Separate regressions were run for each of the predictors while controlling for age and place of birth. Prior to the regression analyses, we first confirmed that the data met all regression assumptions. There was no collinearity between age/place of birth and the X_1_ variables in the model. Additional covariates were not included because of insufficient power (e.g., levels of current health conditions diagnosed by a health professional).

## 3. Results

### 3.1. Sample Characteristics

All participants identified as Hispanic or Latine (100%). The majority identified as: male (57.8%), LGBTQ+ (58.8%), and White Hispanic (54.3%). The majority had completed some college education or a higher academic degree (52.6%), and were born in the United States, Puerto Rico, or the U.S. Jurisdictions (66%). Of those foreign-born (n = 94, 34%), the vast majority were from Mexico (75%) and had lived an average of 18 years in the United States. The sample average age was 36.9 years (SD = 11.9). The largest age groups represented were 25 to 34 years old (39%) and 35 to 49 years old (33%). Levels of current health conditions diagnosed by a health professional included: any anxiety disorder (22.6%), HIV (17.4%), any depressive disorder (20.9%), PTSD (7.3%), any substance use disorder (4.4%), Alcohol use disorder (2.9%), and Hepatitis C (2%).

### 3.2. Barriers to Substance Use and Mental Health Services per Domain

The top perceived personal barriers for substance use and mental health services were, respectively, concerns about disclosure of substance use to government authorities (n = 128, 42.7%) and concerns about people finding out about a mental health issue (n = 148, 49.3%). The top insurance barriers for substance use and mental health services were, respectively, a lack of time to find insurance or insurance benefits due to work or family responsibilities (n = 134, 44.7%) and not having insurance coverage for mental health services (n = 152, 50.7%). The top institutional barriers were that substance use treatment/rehabilitation services are too expensive (n = 123, 41%) and that mental health treatment or therapeutic services are too expensive (n = 135, 45%). The top cultural barriers were feeling that they can manage their alcohol or substance use issue on their own (n = 152, 50.7%) and feeling that they can manage their mental health concerns on their own (n = 131, 43.7%).

The average composite score and range of values for barriers to seeking substance use services per scale were as follows for this sample: perceived personal barriers (Mean = 24.93, SDS = 4.79, Range: 10 to 32); insurance barriers (Mean = 16.05, SD = 3.28, Range: 8 to 20), institutional barriers (Mean = 32.15, SD = 7.8, Range: 14 to 44); and cultural barriers (Mean = 13.03, Sd = 2.58, Range: 4 to 16). The average composite score and range of values for barriers to seeking mental health services per scale were as follows for this sample: perceived personal barriers (Mean = 23.1, SD = 4.5, Range: 9 to 28); insurance barriers (Mean = 16.27, SD = 3.53, Range: 8 to 21), institutional barriers (Mean = 33.13, SD = 8.26, Range: 12 to 44); and, cultural barriers (Mean = 12.07, SD = 2.79, Range: 6 to 16). All the Means of these scales were above the mid-point of each composite scale, with most of the values reported being at the upper tail of the scales, suggesting high levels of significant barriers to seeking substance use and mental health services in the sample.

### 3.3. Influences of Demographic Characteristics on Barriers

Differences in demographic characteristics were associated with differences in reporting multilevel barriers in this sample (see Table 1). Having higher levels of education was significantly associated with perceived personal, institutional, insurance, and cultural barriers to both substance use and mental health services. Female-identifying individuals reported significantly higher numbers of (a) insurance barriers to substance use services, and (b) institutional barriers to mental health services, than those who identified as males. We also found inversed correlations between age reported at the time of the survey: (a) insurance barriers for both substance and mental health services (r = −0.15, *p* < 0.01, and, r = −0.16, *p* < 0.01, respectively); (b) cultural barriers to substance use services, and institutional barriers to mental health services (r = −0.13, *p* < 0.01, and, r = −0.20, *p* < 0.001, respectively). Differences in sexual orientation and place of birth were not statistically associated with differences in any type of barriers (see Table 1).

### 3.4. Influences of Health Background Characteristics on Barriers

In terms of health background variables (see Table 1), we found that respondents with a depression diagnosis reported significantly higher perceived personal barriers to seeking mental health services than their counterparts. Respondents with a PTSD diagnosis reported significantly higher perceived personal, insurance, and cultural barriers to seeking mental health services than their counterparts. Respondents without a substance use disorder diagnosis reported significantly higher institutional barriers to seeking mental health services than their counterparts. HIV negative respondents (without an HIV diagnosis) reported significantly higher institutional barriers to substance use services, and personal, insurance, and institutional barriers to seeking mental health services than their counterparts. No other physical or mental health diagnosis was associated with barriers to substance use services (see Table 1).

### 3.5. Substance Use- and Mental Illness-Related Stigma Levels

Table 2 lists the average substance use and mental illness-related stigma scores per item and their corresponding 95% confidence intervals (estimated population level values). In Table 2, all the average values and intervals are above 2 (Disagree) and below 3 (Agree), suggesting mixed agreement with substance use stigma scale items. Similarly, all the average values and intervals are concentrated around “3” (Agree), suggesting a consistent moderate level of agreement with mental illness stigma scale items.

### 3.6. Associations with Substance Use-Related Stigma

There were no statistical associations between substance use-related stigma scores and multilevel barriers to seeking substance use services (see linear regression models 1 to 4, in Table 3).

### 3.7. Associations with Mental Health-Related Stigma

In this sample, we found that higher mental health-related stigma scores were statistically associated with higher scores in the four types of barriers to seeking mental health services (see linear regression models 5 to 8, Table 3).

## 4. Discussion

### 4.1. Limitations

Our regression models are limited to examining the effects of the main predictors on the stigma-related outcome variables. The role of all predictors jointly while controlling for age and place of birth was not examined in this study. Findings are limited in generalizability to Hispanic/Latine individuals engaged in HIV service organizations in Texas, United States. The cross-sectional design precludes causal inference. Conversely, conducting the study in Texas provided a unique opportunity to explore the role of barriers and stigma in long-term migrant communities in accessing substance use and mental health services.

### 4.2. Community Health Implications

Barriers to substance use and mental health care services focused primarily on concerns about disclosure, cost, and beliefs in self-management in this sample. Moderate-to-high stigma levels highlight the need for stigma-reduction interventions.

### 4.3. Implications for Public Health Science

Contrary to our expectations, we found that substance use-related stigma was not associated with barriers to seeking substance use services. We infer that this could be for two sampling-related reasons. Firstly, the sample had a very low proportion (below 5%) of people reporting an AUD or SUD diagnosis; thus, without an explicit need to seek services, participants are likely not to experience barriers to substance use treatment and care. And yet, because we did not screen for problem use of alcohol or substances (without a diagnosis), it is unknown whether individuals in that group may confront barriers and substance use-related stigma. Secondly, the sample was drawn from HIV prevention and treatment organizations. Historically, HIV services explicitly include substance use early detection, treatment and referral services, ranging from basic education on harm reduction to in-house therapeutic rehabilitation services. Thus, we infer that both substance use-related stigma and barriers to substance use treatment services may have been lower than in non-HIV service-oriented organizations. Additional longitudinal research is needed to examine further the lack of associations found in this study related to substance use stigma and to document the potential role of substance use stigma and multilevel barriers on engagement and retention in substance use care for Hispanic/Latine clients/patients of HIV service delivery organizations.

Our findings also suggest that barriers were reported more frequently amongst those with mental health and substance use diagnoses. This is logical, as additional exposure to the health care system increases the likelihood of experiencing barriers to care. This suggests the importance of mitigating barriers to care amongst those already diagnosed and potentially engaged in care.

In this study, we found that stigma of mental illness was associated with barriers to seeking mental health services. We also found a higher frequency of multilevel barriers to mental health services among HIV negative individuals than among those with an HIV diagnosis. Further research is needed to examine this association (e.g., what are the specific mental health services needs of HIV negative individuals receiving HIV prevention services in community health care settings that increase their likelihood of reporting barriers to mental health care in comparison to those with an HIV diagnosis in the same organizations). This association is concerning, given how mental health is one of the major predictors of HIV acquisition.

## 5. Conclusions

HIV care providers must prioritize the development of targeted strategies that reduce multilevel barriers for key demographic groups: cisgender women or female-identifying; young adults, ages 25 to 34; individuals with higher levels of education than high school; individuals with a diagnosis of PTSD or depressive disorders; and HIV negative individuals. Furthermore, the findings from this study suggest the continuation of support in addressing both multilevel barriers and mental health stigma for recent and long-term Hispanic/Latine immigrant communities engaged by HIV services organizations.

## Figures and Tables

**Table 1:** Barriers by demographic background characteristics among Hispanic/Latine clients/patients (Texas, 2022–2023, n = 300).

	Substance Use Perceived Barriers (Mean Differences)				Mental Health Perceived Barriers (Mean Differences)			
*Characteristic*	*Perceived Personal*	*Institutional*	*Insurance*	*Cultural*	*Perceived Personal*	*Institutional*	*Insurance*	*Cultural*
Gender expression $\bar{x}$**_males–_**$\bar{x}$**_female_**	−0.69	1.07	0.52 *	0.23	−0.28	2.39 *	0.89	0.08
Sexual orientation $\bar{x}$**_LGBTQ+–_**$\bar{x}$**_heterosexual_**	−1.06	−0.24	0.39	−0.25	−0.78	0.51	0.14	−0.43
Education $\bar{x}$**_Some college or higher–_**$\bar{x}$**_High school/vocational or less_**	−2.41 ***	−3.76 ***	−1.82 ***	−1.61 *	−1.77 **	−3.73 **	−1.28 **	−0.79 *
Place of birth $\bar{x}$**_foreign born–_**$\bar{x}$**_US-born_**	1.63	−0.46	0.73	0.32	0.76	0.74	0.57	0.62
PTSD any, lifetime $\bar{x}$**_diagnosed–_**$\bar{x}$**_no-diagnosis_**	1.01	2.53	1.14	0.68	2.32 **	2.26	1.06 *	1.15 *
Depression, any, lifetime $\bar{x}$**_diagnosed–_**$\bar{x}$**_no-diagnosis_**	−0.63	0.22	0.58	0.32	1.44 *	−0.41	0.30	0.43
SUD, any, lifetime $\bar{x}$**_diagnosed–_**$\bar{x}$**_no-diagnosis_**	−1.82	−3.83	−0.47	−0.51	−0.64	−4.84 **	−1.43	−0.34
HIV status $\bar{x}$**_diagnosed–_**$\bar{x}$**_no-diagnosis_**	−1.20	−2.60 *	−0.90	−0.60	−1.90 **	−3.70 **	−1.40 **	−0.50

Notes: (1) Above are Mean differences expressed as subtractions in scores from the subgroup on the left minus the subgroup on the right; (2) Mean differences were tested using independent *t*-tests using Bonferroni test to adjust the *p*-values (* *p* < 0.05; ** *p* < 0.01; *** *p* < 0.001); (3) Regarding health conditions, “diagnosed” means official diagnosis by a medical or health provider; (4) PTSD refers to any post-traumatic stress disorder; (5) Depression refers to any depressive disorder; (6) SUD refers to any alcohol or substance use disorder.

**Table 2:** Average Perceived Stigma of Substance Abuse Scale (PSAS) and Perceived Devaluation and Discrimination Scale (PDD) scores per item, and their corresponding 95% confidence intervals among Hispanic/Latine clients/patients (Texas, 2022–2023, n = 300).

*Outcome Measure*	*n*	*Mean*	*SD*	*95% CI*
**Perceived Stigma of Substance Use (PSAS Items)**				
**Most Hispanics would willingly accept someone who has been treated for alcohol or substance use as a close friend.**	272	2.1	0.89	1.99, 2.21
Most Hispanics believe that someone who has been treated for alcohol or substance use is just as trustworthy as the average person.	282	2.44	0.77	2.35, 2.53
Most Hispanics would accept someone who has been treated for alcohol or substance use as a teacher of young children in a public school.	273	2.63	0.81	2.53, 2.73
Most Hispanics would hire someone who has been treated for substance use to take care of their children.	277	2.84	0.83	2.74, 2.94
Most Hispanics think less of a person who has been in treatment for alcohol or substance use.	272	2.66	0.85	2.56, 2.76
Most employers will hire someone who has been treated for alcohol or substance use if he or she is qualified for the job.	268	2.48	0.8	2.38, 2.58
Most employers will pass over the application of someone who has been treated for alcohol or substance use in favor of another applicant.	262	2.74	0.78	2.65, 2.83
Most Hispanics will be willing to date someone who has been treated for alcohol or substance use.	259	2.18	0.75	2.09, 2.27
**Perceived Stigma of Mental Health Illness (PDD Items)**				
**Most Hispanics believe that a person with a severe mental illness is dangerous and unpredictable.**	278	3.06	0.82	2.96, 3.16
Most Hispanics believe that having a mental illness is worse than using substances.	274	2.97	0.86	2.87, 3.07
Most Hispanics would accept a person with a severe mental illness as a close friend.	264	2.37	0.84	2.27, 2.47
Most Hispanics disparage people after a psychiatric hospitalization.	264	2.92	0.83	2.82, 3.02
Most employers would not hire a person who has been hospitalized for a mental illness.	258	2.89	0.88	2.78, 3.00
Most Hispanics think that people with mental illness are as intelligent as ordinary people.	267	2.55	0.9	2.44, 2.66
Most Hispanics believe that receiving psychiatric treatment is a sign of personal failure.	273	3	0.87	2.90, 3.10
Most Hispanics would not marry a person with a mental illness.	245	2.75	0.94	2.63, 2.87

Notes: (1) Each of the above items were ranked using Likert scale, with the value of “4” representing the highest level of agreement; (2) Mean values represent the average score for the items by the number of respondents to the item; (3) 95% confidence interval for the Mean refer to the range of values calculated from sample data that is likely to contain the true population average based on the sample size.

**Table 3:** Composite barrier scales regressed onto substance use and mental health-related stigma among Latinx clients/patients (Texas, 2022–2023, n = 300).

		PSAS Score β			PDD Score β			Model Significance	
Model	Variable	β	*p*-Value	β_1_ 95% CI	β	*p*-Value	β_1_ 95% CI	F	R^2^
1	Perceived personal (X_1_)	0.11	>0.05	−0.09, 0.21				1.09	0.00
	Age (X_2_)	0.02	>0.05						
	Place of birth (X_3_)	0.04	>0.05						
2	Insurance (X_1_)	0.06	>0.05	−0.01, 0.13				0.72	0.00
	Age (X_2_)	0.03	>0.05						
	Place of birth (X_3_)	0.03	>0.05						
3	Institutional (X_1_)	−0.02	>0.05	−0.21, 0.17				0.01	0.00
	Age (X_2_)	0.06	>0.05						
	Place of birth (X_3_)	0.04	>0.05						
4	Cultural (X_1_)	−0.01	>0.05	−0.07, 0.05				0.04	0.00
	Age (X_2_)	0.01	>0.05						
	Place of birth (X_3_)	0.02	>0.05						
5	Perceived personal (X_1_)				0.66	<0.001	0.53, 0.68	60.06	0.24 ***
	Age (X_2_)				0.04	>0.05			
	Place of birth (X_3_)				0.01	>0.05			
6	Insurance (X_1_)				0.34	<0.001	0.28, 0.41	26.31	0.13 ***
	Age (X_2_)				0.07	>0.05			
	Place of birth (X_3_)				0.03	>0.05			
7	Institutional (X_1_)				0.70	<0.001	0.57, 0.87	17.25	0.09 ***
	Age (X_2_)				0.06	>0.05			
	Place of birth (X_3_)				0.11	>0.05			
8	Cultural (X_1_)				0.25	<0.001	0.21, 0.30	24.13	0.11 ***
	Age (X_2_)				0.05	>0.05			
	Place of birth (X_3_)				0.04	>0.05			

Notes: (1) PSAS refers to Perceived Stigma of Substance Abuse Scale, used as indicator of substance use related stigma for linear regression models 1 to 4, where higher values signify higher agreement; (2) PDD refers to Perceived Devaluation and Discrimination Scale, used as indicator of mental health related stigma for linear regression models 5 to 8, where higher values signify higher agreement; (3) all the models adjusted for age and place of birth; (4) adjusted β-coefficients for predictor variable were reported, with their corresponding 95% confidence intervals; (5) Perceived personal, Insurance, Institutional or Cultural refers to perceived personal, insurance, institutional or cultural barriers to seeking substance use services (models 1 to 4) or mental health services (models 5 to 8); (6) Asterisks indicate the *p*-values (*** *p* < 0.001).

## Data Availability

The data that support the findings of this study are available upon request from the authors.

## References

[1] Pinedo M. (2020). Help seeking behaviors of Latinos with substance use disorders who perceive a need for treatment: Substance abuse versus mental health treatment services. J. Subst. Abus. Treat..

[2] Eghaneyan B.H., Murphy E.R. (2020). Measuring mental illness stigma among Hispanics: A systematic review. Stigma Health.

[3] Turner A., LaVeist A.T.A., Richard P., Gaskin D.J. (2021). Economic Impacts of Health Disparities in Texas 2020.

[4] Callaghan T., Washburn D.J., Nimmons K., Duchicela D., Gurram A., Burdine J. (2019). Immigrant health access in Texas: Policy, rhetoric, and fear in the Trump era. BMC Health Serv. Res..

[5] Hamann J., Bühner M., Rüsch N. (2017). Self-Stigma and Consumer Participation in Shared Decision Making in Mental Health Services. Psychiatr. Serv..

[6] Williams D.R., Lawrence J.A., Davis B.A. (2019). Racism and health: Evidence and needed research. Annu. Rev. Public Health.

[7] Williams D.R., Mohammed S.A. (2013). Racism and health I: Pathways and scientific evidence. Am. Behav. Sci..

[8] West M.L., Yanos P.T., Smith S.M., Roe D., Lysaker P.H. (2011). Prevalence of internalized stigma among persons with severe mental illness. Stigma Res. Action.

[9] Interian A., Ang A., Gara M.A., Link B.G., Rodriguez M.A., Vega W.A. (2010). Stigma and depression treatment utilization among Latinos: Utility of four stigma measures. Psychiatr. Serv..

[10] O’Donnell P., Tierney E., O’Carroll A., Nurse D., MacFarlane A. (2016). Exploring levers and barriers to accessing primary care for marginalised groups and identifying their priorities for primary care provision: A participatory learning and action research study. Int. J. Equity Health.

